# Controllable functionalization and wettability transition of graphene-based films by an atomic oxygen strategy

**DOI:** 10.1007/s11051-013-1811-2

**Published:** 2013-07-02

**Authors:** Min Yi, Wen Zhang, Zhigang Shen, Xiaojing Zhang, Xiaohu Zhao, Yiting Zheng, Shulin Ma

**Affiliations:** 1 Beijing Key Laboratory for Powder Technology Research and Development, Beijing University of Aeronautics and Astronautics, Beijing, 100191 China; 2 Plasma Laboratory, Ministry-of-Education Key Laboratory of Fluid Mechanics, Beijing University of Aeronautics and Astronautics, Beijing, 100191 China; 3 School of Material Science and Engineering, Beijing University of Aeronautics and Astronautics, Beijing, 100191 China

**Keywords:** Graphene, Atomic oxygen, Functionalization, Wettability

## Abstract

Though chemical modification of graphene based on Hummers method has been most widely used to tailor its properties and interfacial characteristics, a method which could achieve definitive and controllable groups and properties is still highly required. Here, we demonstrate a high-vacuum oxidation strategy by atomic oxygen (AO) and investigate the AO induced functionalization and wettability transition in films made from basal-defect- and oxide-free graphene dispersions. These graphene-based films are neither graphene nor graphite, but graphene blocks constituted by numerous randomly stacked graphene flakes. It is found that AO induced functionalization of these films through the formation of epoxy groups, sp^3^ configuration, ether, and double and triple C–O groups. The films turn to be hydrophilic after exposed to AO. The contact angle increases with AO exposure time. This phenomenon is attributed to the lower surface roughness induced by collision and/or edge erosion of energetic ions to the film surface and is further explained by the Wenzel model. The demonstrated strategy can overcome limitations of Hummers method, provide possibility to gain functionalization and wettability transition in liquid-phase exfoliated basal-defect- and oxide-free graphene in the dry environment, and may extend the study and application of this material in spacecraft in low earth orbit.

## Introduction

Graphene, constituted by the two-dimensional honeycomb C–C network, has attracted a great deal of attention due to its exceptional and intriguing properties (Novoselov et al. 2004; Sofo et al. 2007; Geim 2009; Allen et al. 2010; Geim and Novoselov 2007; Tanaka and Iakoubovskii 2010; Rao et al. 2010). Recently, graphene has shown potential applications in lots of fields, such as fundamental physical research (Barone et al. 2006; Katsnelson and Novoselov 2007; Novoselov et al. 2005; Fujita 2011), energy-storage material (Deng et al. 2004; Boukhvalov et al. 2008; Wang et al. 2008), new electronic devices (Novoselov et al. 2004; Lemme et al. 2007; Schedin et al. 2007; Wu et al. 2007; Moriyama et al. 2010; Wakabayashi et al. 2010), catalyses (Sofo et al. 2007; Elias et al. 2009; Zhou et al. 2009), chemical and biological sensors (Schedin et al. 2007; Mohanty and Berry 2008), etc. Ironically, despite its outstanding properties and bright prospects, several issues must be overcome before the full promise of graphene can be realized industrially. For example, graphene is semi-metallic. If graphene is used in microelectronics, chemical modification is required to introduce a band gap to achieve semiconducting behavior (Zhou et al. 2008; Balog et al. 2010; Yavari et al. 2010). In addition, graphene is hydrophobic and chemically inert, making processing and purification techniques difficult. However, in the fields such as catalyses, liquid-phase processing, biocompatibility, etc., graphene is often expected to be chemically active and hydrophilic. Therefore, when graphene is considered in terms of its promising prospect, in order to meet the special requirement in the processing and applications, chemical functionalization of graphene seems very critical and necessary.

Presently, the oxidation technique based on Hummers method is most widely used to covalently functionalize graphene (Hummers and Offeman 1958; Park and Ruoff 2009; Dreyer et al. 2010; Loh et al. 2010). It alters the properties of graphene by introducing some oxygen-containing groups, and the resulting graphene oxide material is highly inhomogeneous. However, Hummers method is often accomplished in the wet solution of strong acids, making it difficult to clearly determine the forming process and precise content of different oxygen-containing groups. Moreover, these oxygen-containing groups cannot be completely removed by chemical or thermal reduction. Hence, these shortcomings limit the applications of the wet chemical functionalization based on Hummers method.

Recently, Elias et al. (2009) reported the functionalization of graphene by atomic hydrogen, which transformed semi-metallic graphene into an insulator. Following this idea, atomic oxygen (AO) may be also a promising agent for chemical functionalization of graphene, because it can forms stable bonds in graphene. Furthermore, AO oxidation happens in the high-vacuum conditions where water or hydrogen (i.e., hydroxyl and acid carboxyl) can be excluded first, making the determination of oxygen-containing groups relatively easy. Moreover, for graphene material as solid films used in electronic and energy applications, direct chemical functionalization in the dry environment is crucial and high-vacuum AO oxidation may provide such a feasible route. We also noted that some researchers investigated the AO induced oxidation of highly oriented pyrolytic graphite (HOPG) surface (Barinov et al. 2009b; Larciprete et al. 2012). In contrast, firstly our work is focused on graphene-based film. Graphene flakes have different properties (especially electronic) compared to bulk HOPG. Most interestingly, the graphene-based film in our work is constituted by numerous graphene flakes which are randomly stacked (not Bernal AB style in bulk graphite). This random stack of graphene flakes has been demonstrated to possess unique band structure and electronic properties. However, presently, there is little information about how to tune the properties of these random stacked graphene flakes. Our work here may provide a way for tuning properties by AO in the dry environment. Second, HOPG surface is flat. The graphene-based film surface is rough with many graphene flakes protruding out, providing possibility for simultaneous functionalization and physical surface engineering. Third, most current studies concentrate on wet chemistry methods to tailor the composition and surface structure of films. We combined AO oxidation and ions’ collisions to tuning the wettability of the graphene-based film in the dry environment.

Meanwhile, some researchers have investigated the oxidation of chemical vapor deposited and epitaxial graphene by AO (Vinogradov et al. 2011; Hossain et al. 2012). However, it is likely that many future industrial applications of graphene depend on large-scale production which could be achieved by liquid-phase method. Recently, Coleman’s group and other researchers did lots of work in this aspect (Hernandez et al. 2008; Coleman 2009; De et al. 2010; Khan et al. 2010, 2011; Lotya et al. 2010; Cui et al. 2011; Shen et al. 2011; Yi et al. 2011; Coleman 2013; Yi et al. 2012a, b). They evidenced that as compared to the methods of micromechanical cleavage, chemical vapor deposition, epitaxial growth, etc., the liquid-phase exfoliation of graphite can obtain basal-defect- and oxide-free graphene and shows advantages of facilitation, large scale, and low cost. Hence, the functionalization of liquid-phase exfoliated basal-defect- and oxide-free graphene material is an alternative way to extend the applications of this material. Yet, to our knowledge, though there are many reports on covalent functionalization by organic groups or noncovalent functionalization by π-interactions of liquid-phase exfoliated pristine graphene in the wet environment (Georgakilas et al. 2012), there is little information about functionalizing liquid-phase exfoliated pristine graphene in the dry environment.

**Fig. 1:**
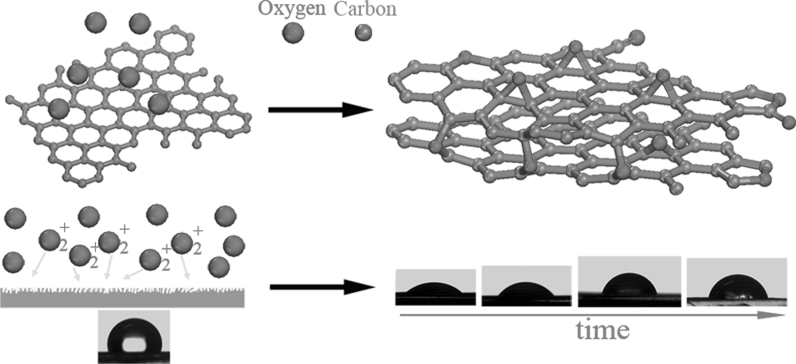
Schematic image of AO induced controllable functionality and wettability transition. In the AO oxidation strategy, AO induced functionalization facilitates the determination and control of oxygen-containing groups in graphene and turns graphene-based films from hydrophobic to hydrophilic. Longer collision and/or edge erosion of energetic ions onto the film surface can achieve less rough surface and thus result in larger contact angles

Herein, we prepare films by vacuum filtering the dispersions of basal-defect- and oxide-free graphene and investigate the oxidation process of these films exposed into AO with different time. On the other hand, in spite of intensive activity in graphene research, there are very few literatures about water–graphene interactions (Wang et al. 2009; Rafiee et al. 2010; Yavari et al. 2010; Dhiman et al. 2011; Lin et al. 2011; Choi and Park 2012; Nair et al. 2012). Most current studies concentrate on wet chemistry methods to tailor the composition and surface structure of films. Herein, we utilize the energetic ions in the AO atmosphere to collide with the film surface and investigate the wettability transition behavior of these films under different AO exposure time. Figure 1 illustrates the process of AO induced controllable functionality and wettability transition in graphene-based films. In a word, the demonstrated strategy of AO oxidation provides possibility to gain functionalization and wettability transition of basal-defect- and oxide-free graphene from liquid-phase exfoliation in the dry environment, and may extend the study and application of this material. The study here is also very valuable for graphene-based electronics and sensors to be used in spacecraft in low earth orbit where AO and energetic ions abound.

## Experimental

### Preparation and characterization

Dispersion of basal-defect- and oxide-free graphene was prepared by liquid-phase exfoliation of graphite (Hernandez et al. 2008; Coleman 2009; Khan et al. 2010, 2011; Coleman 2013). Graphite dispersions were made by 70 mg crystal graphite powder (≤300 meshes, Alfa Aesar) dispersed in 140 mL dimethylformamide (DMF). These dispersions were then sonicated for 60 min in a low-power ultrasonic bath (45 W, 59 Hz, SK1200H-J, KUDOS, Shanghai). After sonication, the dispersions were centrifuged for 30 min at 2,000 rpm (570×*g*) by an L-600 centrifuge (XiangYi, Changsha) in order to remove any largish flakes, eventually resulting in homogeneous colloidal suspension of graphene flakes in DMF. Graphene concentration after centrifugation, *C*_G_, was determined from Lambert–Beer law, *A*/*l* = *αC*_G_, where *A* is the absorbance measured at 660 nm by a 721E spectrophotometer (Shanghai Spectrum), *l* is the cuvette length, and *α* is absorption coefficient at 660 nm which equals to 2,460 mL/mg/m (Hernandez et al. 2008, 2010). By using this method, the concentration of the resulted graphene dispersions can be determined as ~45 μg/mL. Considering an initial concentration of 0.5 mg/mL (70 mg/140 mL), we can estimate a yield of ~9 wt%.

50 mL graphene dispersions were diluted to 1,000 mL (50 mL for preparing the loose film) by deionized water and then vacuum filtered to form a homogeneous film (Φ 40 mm) on the nylon membrane (pore size ~220 nm). The film was dried for 48 h in the vacuum oven at 100 °C. Subsequently, a 30 × 20 mm rectangular small film was cut from the film central region. Atomic force microscopy (AFM) was used to scan 96 points on the surface of the small film to determine the roughness. Average of the surface roughness (root-mean-square roughness) corresponding to each scan was used as the surface roughness of the small film. The small film was again cut into 6 bitty films with dimension of 10 × 10 mm. One bitty film was chosen to measure the contact angle with water. The other 5 bitty films were exposed into high-vacuum AO atmosphere with time of 3, 5, 10, 20, and 25 h. After AO exposure, the surface roughness and contact angle of these films were measured, and X-ray photoelectron spectroscopy (XPS) and Fourier transform infrared (FTIR) spectroscopy analyses were performed. When the roughness of the 10 × 10 mm films was measured by AFM, 16 points were scanned in each sample and the average surface roughness was adopted.

AFM images were captured by a CSPM5500 AFM (Being Nano-Instruments) with a scanning range of 13.56 μm in the tapping mode. Bright-field transmission microscope (TEM) and high resolution TEM (HRTEM) images were taken with a JEOL 2100FEF operating at 200 kV. When AFM and TEM were used to characterize the morphology and structure of the isolated or individual graphene flakes, AFM samples were prepared by spraying several microliters of dispersions onto the mica substrate and dried in vacuum oven, while TEM samples were prepared by pipetting several drops of dispersions onto holey carbon mesh grids (300 meshes). The Raman measurements were made on these films with a Renishaw Rm2000 using a 514 nm laser. FTIR spectra of the films were measured by a Nicolet iS10 spectrometer in the diffuse reflection mode. XPS investigation was performed on the films by an ESCALAB-250 photoelectron spectrometer (Thermo Fisher Scientific) with the monochromatic Al Kα X-rays source (1,486.6 eV). For each spectrum, the binding energy position was calibrated by measuring the Fermi level position of an Au reference sample in contact with graphene-based film. Static contact angle was measured by placing a droplet of deionized water on the surface of the various films. An Easydrop DSA20 instrument was used to measure the contact angle at room temperature (~25 °C). The axisymmetric-drop-shape analysis profile method was used for estimating the contact angle of water on the film surface.

### AO exposure experiment

**Fig. 2:**
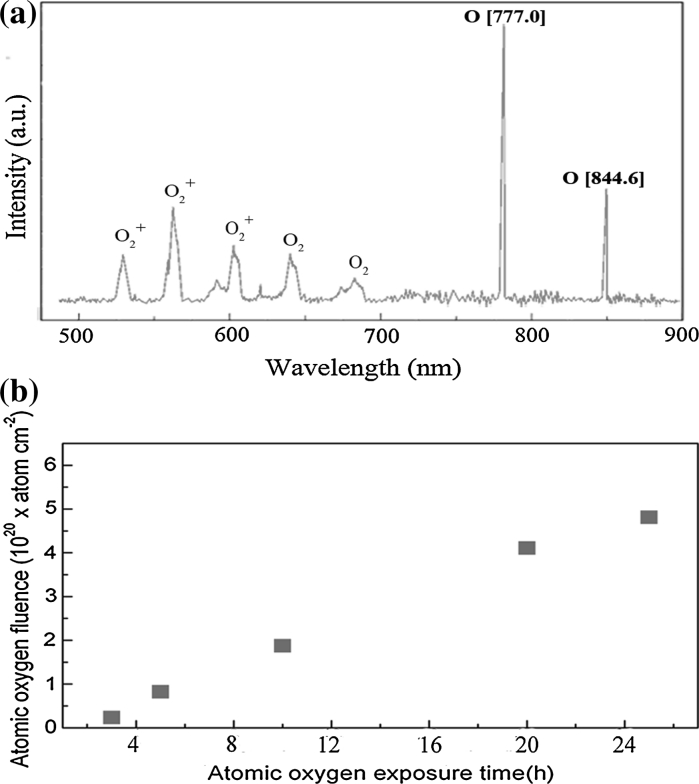
**a** Oxygen plasma emission spectrum. **b** AO fluence varying with time

High-vacuum AO functionalization of these bitty films was performed in the ground-based AO effect simulation facility in Beijing University of Aeronautics and Astronautics (BUAA) (Zhao et al. 2001). The facility was a filament discharge plasma-type ground-based AO effect simulation facility for simulating low earth orbit environment (Zhao et al. 2001). The filament was heated to a high temperature by current. When the temperature is high enough, electrons could escape from the filament and collide with the oxygen molecules, making oxygen molecules ionized into oxygen plasma. The main components of the plasma include O_2_, O_2_^+^, O, O^+^, e, etc., as shown in Fig. 2a. It has been evidenced that the energy of AO was only ~0.04 eV while the energy of O_2_^+^ could be as high as 15 eV (Zhao et al. 2001). AO exposure experiments were carried out in the conditions of vacuum pressure of 0.15 Pa, filament discharge voltage of 120 V, and filament discharge current of 140 mA. Because the erosion yield of Kapton under AO almost keeps constant, the mass loss of Kapton in the AO exposure experiment was used as a criterion to calculate the AO flux (Reddy et al. 1993; Zhao et al. 2001; Banks et al. 2006). The calculation formula is Ft = *ΔM*/(ρ*AE*_y_) in which *F* is the effective flow rate of AO onto the sample surface and *ΔM*, *ρ*, *A*, *t*, *E*_y_ are mass loss, density, surface area, exposure time, and erosion yield, respectively. Ft is the accumulative AO flux exerting on the sample surface. For Kapton, *E*_y_ is equal to ~3.0 × 10^−24^ cm^3^/atom (Zhao et al. 2001; Banks et al. 2006), so according to the mass loss of Kapton, the accumulative AO flux for the films can be obtained, as indicated in Fig. 2b.

## Results and discussion

Though micromechanical cleavage and chemical vapor deposition can achieve high-quality graphene, they have limitations in low cost and scalable production. Methods based on graphene oxide makes low-cost and large-scale production possible, but they often obtain graphene-like material with defects which cannot be completely removed. However, many further industrial applications of graphene require a method which can prepare high-quality graphene with low cost and large quantities. Recently, many works show that liquid-phase exfoliation of graphite is a viable method to prepare basal-defect- and oxide-free graphene with low cost and large quantities (Hernandez et al. 2008, 2010; Coleman 2009; Khan et al. 2010, 2011; Lotya et al. 2010; De et al. 2010). Nevertheless, the functionalization of basal-defect- and oxide-free graphene prepared by this method is rarely studied currently. While for some special requirements in the research and applications of this material, functionalization is indispensable. Therefore, we demonstrate a strategy for utilize high-vacuum AO to tailor the functionalization and wettability transition of this kind of graphene material.

**Fig. 3:**
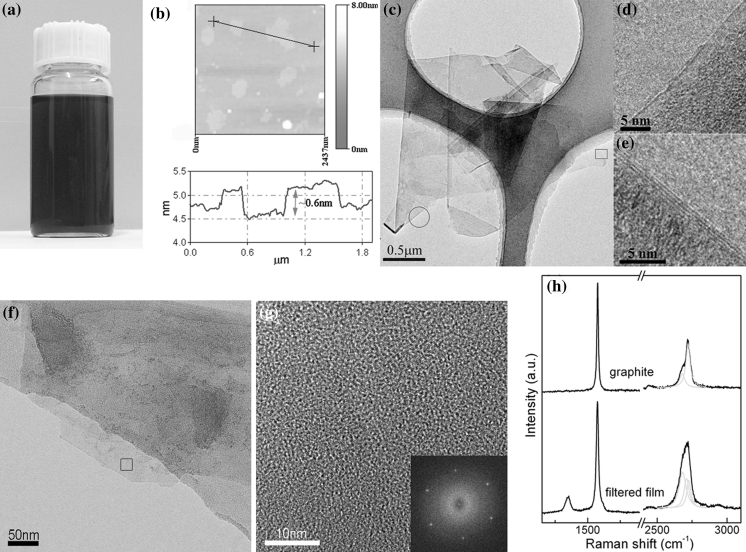
**a** A photograph of the prepared graphene dispersions. **b** A typical AFM image of several graphene flakes with a height profile showing the graphene thickness. **c** Bright-field TEM image of typical folded and piled graphene flakes. HRTEM images of the flake edges indicating graphene flakes with thickness of **d** single (the *rectangle* in **c**) and **e** three (the *circle* in **c**) layers. **f** Bright-field TEM image of a graphene flake. **g** HRTEM image of the* square* region in **f**. *Inset* FFT (equivalent to an electron diffraction pattern) of the *square* region in **f** showing typical sixfold symmetry. **h** Raman spectra of pristine graphite and the filtered film made from graphene dispersions

First of all, we investigated the quality of the prepared graphene flakes in graphene dispersions presented in Fig. 3a. Figure 3 gives the AFM, TEM, and Raman spectra results. The individual graphene flakes captured by AFM in Fig. 3b are with thickness of ~0.6 nm and lateral size of several 100 nm. According to the single-layer thickness of ~0.345 nm (Novoselov et al. 2004), these flakes can be deemed as single layer because a bilayer is at least 0.69 nm thick. Figure 3c presents the typical TEM image, in which graphene flakes are often folded and piled. The flakes captured by TEM are with lateral size of several micrometers, often larger than those captured by AFM. This could be attributed to that the smaller flakes may be lost through the holes in the grid used for TEM samples. Figure 3d, e gives the HRTEM images of the folded edges in Fig. 3c. According to the fringe contrast at the edge (Ferrari et al. 2006; Shen et al. 2011; Yi et al. 2011), the layer number of graphene in the rectangle and circle region in Fig. 3c can be determined as one and three, respectively. Shown in Fig. 3f is a bright-field TEM image of a graphene flake. Fig. 3g gives the HRTEM image of the square region in Fig. 3f. The inset of Fig. 3g, a fast Fourier transform (FFT) of this image which is equivalent to a diffraction pattern, shows typical sixfold symmetry as expected in graphene structure. Furthermore, we investigated the structure and defect information of the filtered film before AO oxidation. Figure 3h gives the Raman spectra of pristine graphite powder and the filtered film. Obviously, narrow and weak D peak in the filtered film is intrinsically different from the wide and intensive D peak in the widely investigated reduced graphene from graphene oxide (Li et al. 2008; Tung et al. 2009; Stankovich et al. 2006, 2007). And G peak in the filtered film shows no apparent widening as compared to that in the pristine graphite powder. Hence, the possibility that D peak in the filtered film is induced by basal defects is extremely low; because basal defects could induce G peak’s apparent widening which is often found in the chemically reduced graphene (Stankovich et al. 2006, 2007; Hernandez et al. 2008; Li et al. 2008), while G peak in the filtered film here is not widened. In addition, the introduction of edge defects is unavoidable; because cavitation-induced shear force and shock waves cut the initial large crystallite into smaller flakes and the dynamic flow during the vacuum filtration may tear or fold micrometer sheets into submicrometer ones (Suslick and Price 1999; Cravotto and Cintas 2010; Shen et al. 2011). These smaller flakes in the filtered films have more edges per unit mass so that increase the content of edge defects. Consequently, seeing that the broadening of G band is unremarkable and the size of laser point (1–2 μm) used in the Raman system will inevitably cover the edges of graphene sheets in the filtered film, the D band in the filtered film may be largely attributed to the edge defects instead of the basal defects. We can also look at the 2D band. The 2D band in the filtered film is apparently distinct from 2D band in pristine graphite, indicating the nature of few-layer graphene (Ferrari et al. 2006; Hernandez et al. 2008; Malard et al. 2009). This indicates that though aggregation of graphene flakes happens during the filtration, the aggregation is not a process to drive graphene flakes stacked in Bernal AB style which exists in graphite. Therefore, the filtered film is neither graphene nor graphite, but a graphene block constituted by numerous graphene flakes which are randomly stacked.

**Fig. 4:**
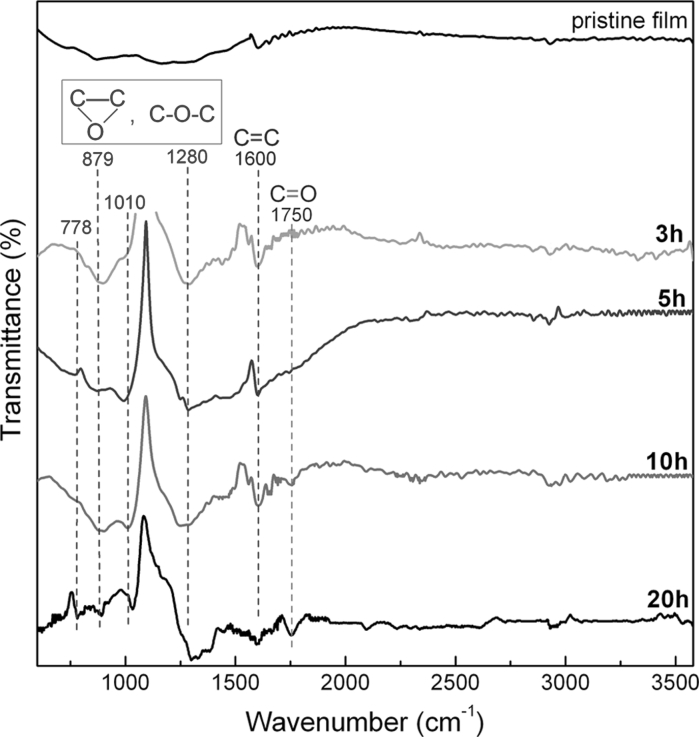
FTIR spectra of the pristine film and films with different AO exposure time

**Fig. 5:**
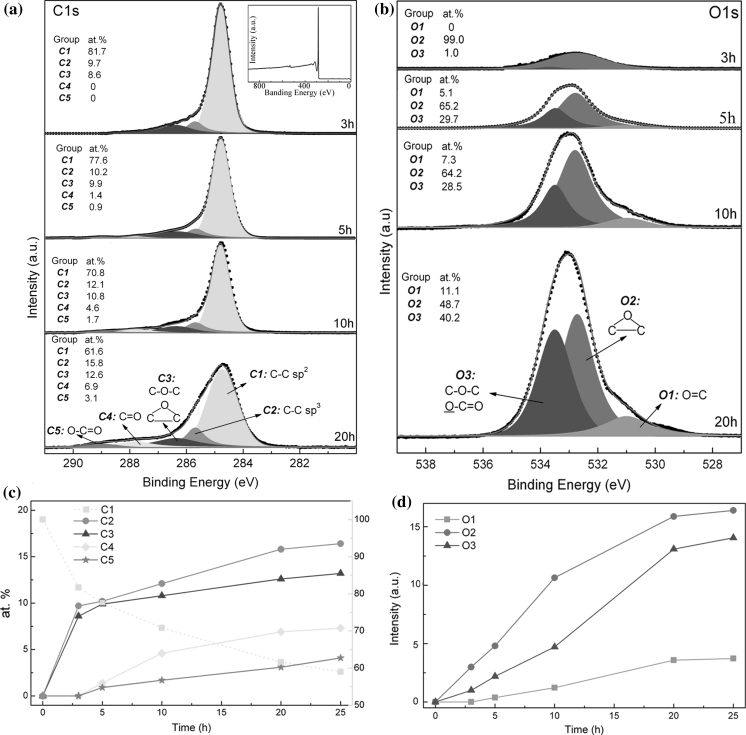
Deconvolution of the **a** C1s and **b** O1s wide spectra of films after AO exposure with different time. *Inset* of **a** shows the XPS spectrum of the pristine film. The intensity of C1s spectrum in 20 h is magnified by a factor of four to clearly present the detail information. O–C=O means the etheric oxygen of the carboxyl group. **c** Atomic percent of C1s components versus AO exposure time. The *dotted line* in **c** refers to the right axis. **d** Intensity of O1s components versus AO exposure time

Figures 4 and 5 give the FTIR and XPS results of the films before and after AO exposure. The FTIR of the pristine film in Fig. 4 shows no peaks associated with oxygen-containing groups. The inset XPS spectrum in Fig. 5a also shows predominant C1s peak and unobservable O1s peak. These results further verify that the prepared graphene and thus the film are largely free of basal defects and oxide, as reported by the Coleman group (Hernandez et al. 2008; Coleman 2013). It can be seen from Fig. 4 that peaks associated with oxygen-containing groups appear in the FTIR after AO exposure. Because oxidation happens in the high-vacuum condition where water or hydrogen does not exist, the formation of hydroxyl and acid carboxyl can be excluded. The absorption peaks at 778, 879, 1,010, and 1,280 cm^−1^ are attributed to the formation of ether and/or epoxy groups (Hontoria-Lucas et al. 1995; van Dijk-Wolthuis et al. 1995; Mao and Gleason 2004; Titelman et al. 2005; Wang et al. 2009). The determination of whether ether or epoxy forms in the different exposure time needs further XPS analyses. Meanwhile, it is clear that the C=O group (Bagri et al. 2010; Hu et al. 2012) at ~1,750 cm^−1^ does not presents observable peak until the AO exposure time is over 10 h. This indicates the C=O group forms at the deep oxidation stage. All the peaks in FTIR spectrum (Fig. 4) evidence that AO can react with the film to form different oxygen-containing groups. In addition, by weighting the film mass, it was found that the mass of each film nearly keeps constant during the whole experiment. This is entirely different from the case of polymers which are often used in spacecraft and can react with AO to form volatile gas and induce severe mass loss (Reddy et al. 1993; Zhao et al. 2001; Banks et al. 2006; Bitetti et al. 2007). Thus, it can be speculated that AO oxidation of these films only generates oxygen-containing groups in the graphene flakes, without gas formation.

In order to ascertain the formation process of oxygen-containing groups during AO oxidation, we performed XPS analysis, as shown in Fig. 5. According to the well-established relationship between C1s components and possible oxygen-containing groups forming during oxidation (Zielke et al. 1996; Ago et al. 1999; Ionescu et al. 2006; Larciprete et al. 2009), fitting C1s spectra into several components contributes to reveal the groups forming process. Based on the experience of analyzing functional groups of carbon material by XPS (Zielke et al. 1996; Ago et al. 1999; Ionescu et al. 2006; Larciprete et al. 2009), we can fit the C1s spectrum with five peaks using banding energies of ~284.80 (C1), ~285.69 (C2), ~286.37 (C3), ~287.78 (C4), and ~289.00 eV (C5), as show in Fig. 5a. C1 and C2 are all related to C–C bond. C1 exists in the whole experiment and its binding energy is the lowest, so we can attribute C1 to the undisturbed sp^2^ carbon in graphene. C2 at ~285.69 eV has been reported in the research about the oxidation of graphite material and is attributed to the sp^3^ carbon (Ago et al. 1999; Estrade-Szwarckopf 2004; Larciprete et al. 2009). Also, because of structural disorder induced by oxidation, C1 (sp^2^ C) component widens with increasing exposure time. These indicate that AO functionalization can disturb sp^2^ hybridization and induce sp^3^ hybridization in graphene. C3, C4, and C5 are attributed to single C–O–C bonds (epoxy, ether), double C=O bond, and triple O–C=O bond. In the case of O1s spectra, C=O groups often appear between the binding energy of 531 and 532 eV and C–O bonds in ether and hydroxyl appear between 533.8 and 534.6 eV (Zielke et al. 1996; Rjeb et al. 2000). Based on these considerations and the results of thermal decomposition of different oxygen-containing groups under different temperature (Zielke et al. 1996; He et al. 1998; McAllister et al. 2007; Barinov et al. 2009a), we have best-fitted the O1s spectra with three components at ~531.0 eV (O1), ~532.7 eV (O2), and ~533.5 eV (O3). These components correspond to double C=O bond, single epoxy bond, and single ether (C–O–C) and etheric oxygen of the carboxyl group (O–C=O), respectively.

According to the above analyses of C1s and O1s spectra, Fig. 5c gives the atomic percentage of different oxygen-containing groups in C1s spectra as a function of AO exposure time. Figure 5d gives the O1 s component intensity (peak area) varying with AO exposure time. Epoxy could disturb the pristine sp^2^ carbon in graphene and induce sp^3^ hybridization in the C atoms neighboring epoxy. So C2 components related to sp^3^ C also appear. The formation of ether groups may be attributed to three reasons: AO reacts with point defects in the basal plane of graphene; AO reacts with edge carbons; AO penetrates from edge and intercalates between randomly stacked graphene flakes to react with adjacent two carbon atoms from different flakes. The above Raman, FTIR, and XPS analyses of the pristine film have confirmed that the prepared graphene is largely free of basal defects and the film is constituted by numerous randomly stacked graphene flakes. Large quantities of edges and disorder stacks exist in the film. So the possibility of the first reason for ether formation is extremely low, and the formation of ether may be mainly attributed to the second and third reasons. Recently, some researchers investigated the oxidation of single-layer graphene prepared by chemical vapor deposition. They also found that AO can penetrate under the graphene from the graphene grain boundary and intercalate between the metal substrate and graphene to form ether-like groups (Vinogradov et al. 2011). This further evidences the existence of the above-mentioned third reason. Based on the above discussion, the strategy of AO functionalization shows advantages over the most widely used Hummers method, because wet chemistry strategy of functionalizing graphene based on Hummers method often introduces miscellaneous groups including hydroxyl and acid carboxyl and the type and content of these groups are very difficult to tune effectively. In this aspect, functionalization of graphene based on AO provides possibility to tailor the functional properties of graphene material made from liquid-phase exfoliated graphene.

**Fig. 6:**
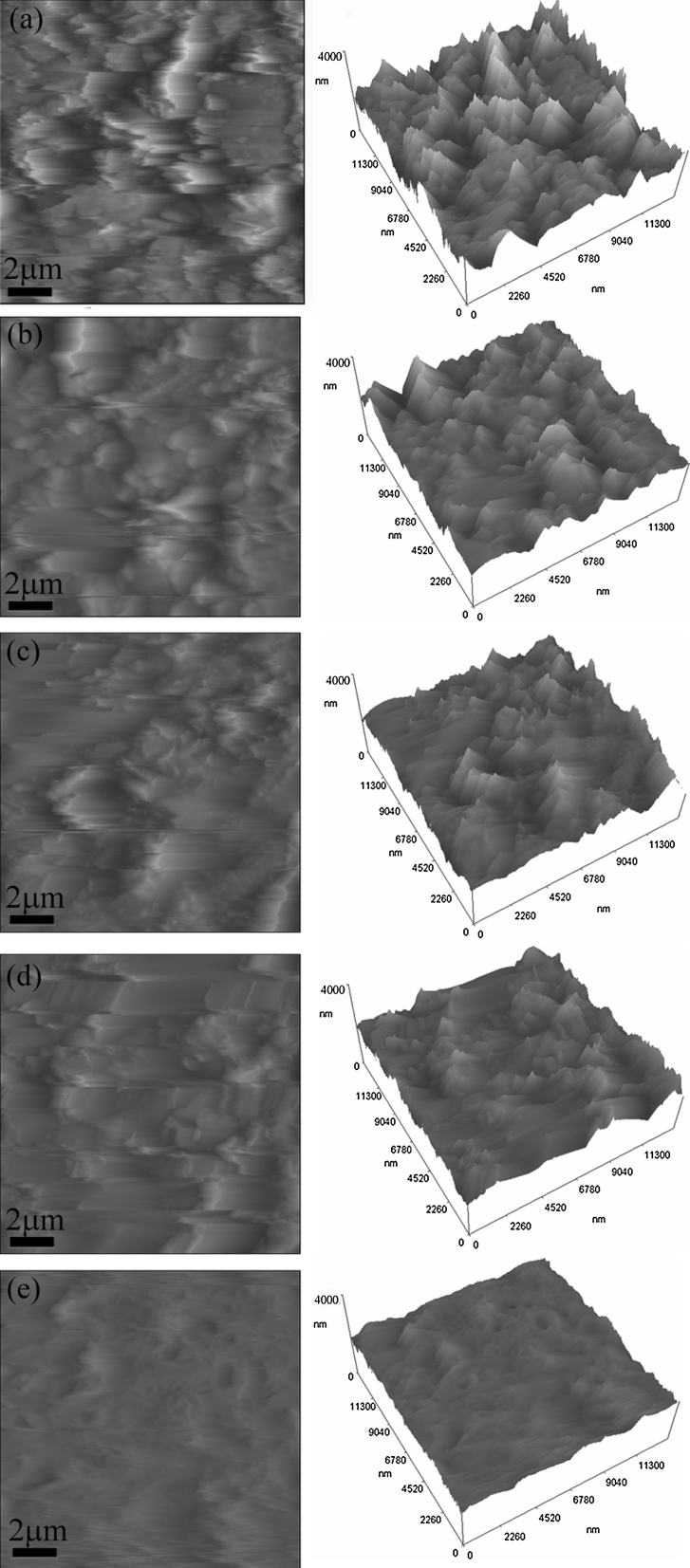
Typical AFM images of the pristine film (**a**) and films with AO exposure time of **b** 3 h, **c** 5 h, **d** 10 h, and **e** 20 h. *Scale bar* 4,000 nm

**Fig. 7:**
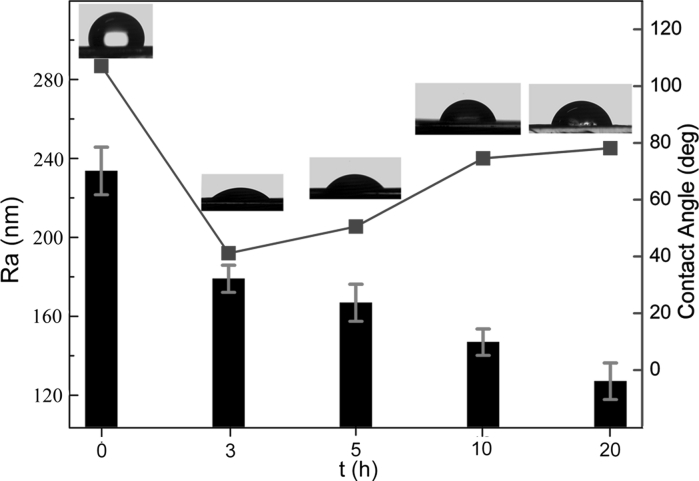
Surface roughness (root-mean-square roughness, *R*_*a*_) and contact angles of the pristine film and films with different AO exposure time

On the other hand, the study on interactions between water and graphene material is also critically important for applications of graphene in conformal coatings. But currently there are very few works in this aspect, and most investigation concentrates on tailoring wettability by wet chemistry based methods. For solid films used in electronic devices or energy storage, their direct functionalization in dry environment is crucial and high-vacuum AO oxidation may provide such a viable route. With these considerations, we further investigated the wettability transition of these prepared films. Because the wettability of a film is closely related to the surface roughness, we firstly used AFM to investigate the surface morphology of these films and further to determine the average surface roughness (*R*_a_, root-mean-square roughness). Figure 6 gives the typical 2D and 3D AFM images of these films’ surfaces. It is obvious that graphene flakes and their edges vertically protrude toward the surface in the pristine film, making a very rough surface, as shown in Fig. 6a. Nevertheless, as the AO exposure time increases, the film surfaces tend to be more flat, as shown in Fig. 6b–e. This phenomenon may be attributed to the collision of energetic ions onto the film surface. It should be noted that our filament discharge plasma-type ground-based AO effect simulation facility was designed for simulating low earth orbit environment (Zhao et al. 2001). Because the AO generated in our facility only has erosion effects, we use the collision of energetic ions to simulate the collision of AO in the real low earth orbit (Zhao et al. 2001). The energetic ions in our facility mainly contain O_2_^+^ and their energy is about 15 eV (Zhao et al. 2001). Though some researchers have shown that energetic ions with energy of several to several hundred KeV could induce defects or react with graphene (Ugeda et al. 2010; Akcoltekin et al. 2011; Bubin et al. 2012), we still have no solid evidence that ions of such a low energy (15 eV) would not react with graphene in the experiment here. So some surface erosion due to the action of the energetic ions (15 eV) cannot be excluded. This maybe occurs when the graphene surfaces are exposed directly to the O plasma. Such a process would erode predominantly the edges protruding out of the surface and this could be the reason for the roughness smoothing revealed in the AFM images as following. The other possibility is that, these ions can exert physical collision on the film surface and cause changes in the surface roughness. In order to prove this speculation, we directly used 50 mL graphene dispersions to prepare a film by vacuum filtration. This film is very loose, because the dispersions volume is so small that the filtration time is too short to obtain a compact film by the dynamic flow during vacuum filtration. It was found that black graphene flakes in the loose film disappeared and only white substrate membrane remained just after AO exposure of 2 h. However, as mentioned above, the compact films prepared by diluted 1,000 mL dispersions kept constant in mass during the whole experiment (25 h). So these analyses confirm that energetic ions in AO atmosphere indeed collide with the film surface. During the collision, graphene flakes and their edges which vertically protrude toward the surface would tend to be parallel with the surface, thus making roughness surface more flat. We further determine the roughness of every film sample by averaging the roughness values from 16 AFM scans in each sample, as shown in Fig. 7 where the error bars are the standard derivation relative to the average roughness. Apparently, the film surface roughness decreases with the AO exposure time, in accordance with the results in Fig. 6.

Furthermore, we measured the contact angles of these films, as shown in Fig. 7. The contact angle of the pristine film is ~107°, indicating hydrophobic nature of graphene. However, after AO exposure, these films turn to be hydrophilic. The wettability transition is attributed to the AO induced functional groups in graphene which increase the surface energy, as evidenced in FTIR and XPS results. Most interestingly, when the films are hydrophilic, the contact angles increase with the exposure time, as shown in Fig. 7. To explain these results, we consider the Wenzel model (Wenzel 1936, 1949) which describes the wetting behavior of water drops on the rough surface. In the Wenzel model (Wenzel 1936, 1949), the apparent contact angle on a rough surface, *θ*_*w*_ is expressed as:

1 $$ { \cos }\theta_{w} = r{ \cos }\theta $$

where *θ* is the contact angle on the ideal flat surface and *r* is the roughness ratio which is defined as the ratio of the true area of the solid surface to its projection area. Since *r* is always larger than 1 for rough surface, according to the expression (1), *θ*_*w*_ is lower than *θ* and decreases with *r* when a surface is hydrophilic (*θ* < 90°). Therefore, as the AO exposure time increases, the decreasing surface roughness would lead to a decreasing *r* and thus an increasing contact angle *θ*_*w*_, as shown in Fig. 7. So the Wenzel model can perfectly explain these results.

For a more detailed analysis, examining the correlation among surface content of the O-carrying groups, morphology roughness, and wettability (contact angle), we can find that as AO exposure time increases, the O-containing groups content increases (Fig. 5c, d), indicating an increase in surface energy. This would result in a decrease in contact angle with water. This may indicate that the wettability transition is indeed attributed to the AO induced functional groups which increase the surface energy. However, after hydrophilicity has been achieved, we experimentally found an increase in contact angle (Fig. 7). From expression (1), if *θ* holds constant and *r* decreases, *r*cos*θ* will decrease and thus *θ*_*w*_ will increase. If *r* holds constant and *θ* decreases, *r*cos*θ* will increase and thus *θ*_*w*_ will decrease. So *r* and *θ* have opposite effects on *θ*_*w*_. The competition between *r* and *θ* will determine whether *θ*_*w*_ is decreased or not. The results on experimentally measured contact angle (Fig. 7) show that as AO exposure time increases, *r* decreases while *θ*_*w*_ increases. This indicates that though *r* decreases and *θ* decreases, the product *r*cos*θ* decreases and thus leads to an increasing trend of *θ*_*w*_ which varies with AO exposure time. Hence, it is possible that once the graphene-based film has become hydrophilic, the hydrophilic level (contact angle) will be predominately determined by the Wenzel model in the scope of surface physics (surface roughness) not surface chemistry (surface functionalization).

## Conclusions

In conclusion, we have demonstrated a high-vacuum oxidation strategy by AO and investigated the AO induced controllable functionalization and wettability transition in graphene-based films which are made from defect- and oxide-free graphene dispersions. These films whose surface roughness can be tailored are neither graphene nor graphite, but a randomly stacked graphene blocks which are constituted by a large number of graphene flakes in disordered arrays. High-vacuum oxidation conditions facilitate the determination and control of oxygen-containing groups. It is found that AO induced functionalization of these films through the formation of epoxy groups, sp^3^ configuration, ether, and double and triple C–O groups. AO induced functionalization turns these films from hydrophobic to hydrophilic. The collision and/or edge erosion effects of energetic ions in the AO atmosphere can lower the film surface roughness, and thus result in larger contact angles which can be explained by the Wenzel model. The demonstrated high-vacuum oxidation strategy shows many advantages over Hummers method in controlling and determining functional groups. This strategy provides possibility to gain functionalization and wettability transition in liquid-phase exfoliated basal-defect- and oxide-free graphene in the dry environment. The study is also very valuable for graphene-based electronics and sensors to be used in spacecraft in low earth orbit where AO and energetic ions abound.
